# Mental Health Impacts of the COVID-19 Pandemic on International University Students, Related Stressors, and Coping Strategies

**DOI:** 10.3389/fpsyt.2020.584240

**Published:** 2020-11-23

**Authors:** Agnes Yuen-kwan Lai, Letitia Lee, Man-ping Wang, Yibin Feng, Theresa Tze-kwan Lai, Lai-ming Ho, Veronica Suk-fun Lam, Mary Sau-man Ip, Tai-hing Lam

**Affiliations:** ^1^School of Nursing, The University of Hong Kong, Pokfulam, Hong Kong; ^2^School of Chinese Medicine, The University of Hong Kong, Pokfulam, Hong Kong; ^3^School of Health Science, Caritas Institute of Higher Education, Hong Kong, Hong Kong; ^4^School of Public Health, The University of Hong Kong, Pokfulam, Hong Kong; ^5^Department of Medicine, The University of Hong Kong, Pokfulam, Hong Kong

**Keywords:** mental health, stress, anxiety, depression, insomnia, students, university, coping

## Abstract

**Background:** The coronavirus disease 2019 (COVID-19) pandemic has disrupted university teaching globally. The mental health impacts on international university students have been overlooked.

**Aims:** This study examined the differences in COVID-19-related stressors and mental health impacts between international university students studying in the UK or USA who returned to their home country or region (returnees) and those who stayed in their institution country (stayers), and identified COVID-19-related stressors and coping strategies that were predictors of mental health.

**Method:** An online questionnaire survey was conducted from April 28 through May 12, 2020 using an exponential, non-discriminative snowball sampling strategy (registered at the National Institutes of Health: NCT04365361).

**Results:** A total of 124 full-time international university students (36.3% male) were included: 75.8% had returned to their home country or region for reasons related to COVID-19; 77.4% were pursuing a bachelor's program, and 53.2% were in programs with practicum component. 84.7% of all students had moderate-to-high perceived stress, 12.1% had moderate-to-severe symptoms of anxiety and depression, and 17.7% had moderate-to-severe symptoms of insomnia. Compared with returnees, stayers had significantly higher stress from COVID-19-related stressors such as personal health and lack of social support (Cohen's d: 0.57–1.11), higher perceived stress [10-item Perceived Stress Scale (PSS-10)] {22.6 ± 6.2 vs. 19.1 ± 6.1, β [95% confidence interval (CI)]: 4.039 (0.816, 7.261), Cohen's d: 0.52}, and more severe insomnia symptoms [Insomnia Severity Index (ISI)] [11.8 ± 6.1 vs. 7.6 ± 5.2, β (95% CI): 3.087 (0.262, 5.912), Cohen's d: 0.45], with moderate-to-large effect sizes. Compared with males, females reported significantly higher stress from uncertainties about academic program (Cohen's d: 0.45) with a small effect size. In the total sample, stress related to academics (e.g., personal attainment, uncertainties about academic program, and changes in teaching/learning format), health (including personal health and health of family and friends), availability of reliable COVID-19-related information, and lack of social support predicted more negative mental health impacts. Resilience, positive thinking, and exercise were predictors of less severe mental health impacts.

**Conclusions:** Stayers experienced more adverse mental health impacts than returnees. We call on educators and mental health professionals to provide appropriate support for international students, particularly the stayers, during the pandemic.

## Introduction

The coronavirus disease 2019 (COVID-19) pandemic has aroused fear and anxiety globally, which may lead to an upsurge in the incidence and severity of mental health problems (1). Global attention has largely focused on infected patients and frontline health workers. Our PubMed search on June 26, 2020 using keywords including “international students,” “mental health,” “pandemic,” “epidemic,” and “outbreak” yielded a limited number of articles on the mental health impacts of COVID-19 in local students (2–4). We found one correspondence piece on the need for mental health care for Chinese international students and one qualitative article on 28 Chinese international students' health risk perception during travel (5, 6). However, there were no articles focused specifically on the mental health impacts of COVID-19 on international students; this group's mental health has been overlooked.

Many universities around the world have implemented preventive measures, including closing campuses or facilities, canceling classes, transitioning to online-based teaching/learning curriculum and examinations, and postponing practicums. However, up to now (mid-June 2020), many universities are still uncertain about how long such measures will continue, and it is unclear how these changes have affected students. Such disruptions due to the COVID-19 pandemic can exert unique additional pressures, adversely affecting students' mental health, with impacts including increased stress, anxiety, and depression (3, 4). In general, university students face a wide range of transitional events and ongoing stressors while adapting to new academic environments and demands. Ongoing stress can affect academic performance as well as mental well-being (7). Such stress may have a disproportionate impact on females compared with males. It has been demonstrated that stress exposure during puberty has stronger proximal effects on girls, including increased risks of developing mood- and stress-related disorders, such as depression, anxiety, and posttraumatic stress disorder (8). More psychological support from academic institutions is needed to enhance female students' mental health and resilience.

For international students, living abroad, adjustment to the host country's culture and norms, and being away from central social support systems such as family and friends can be additional challenges that affect mental health. Students from different countries may have different cultural characteristics, which might affect their coping strategies (9).

During the early stages of the outbreaks in the UK and USA (March 2020), publicly available information and recommendations were often unclear or conflicting. For example, while wearing face masks was not initially advised as a preventive measure, the international recommendations regarding masks subsequently changed. International students from Asia (e.g., students from Hong Kong) might have experienced conflict because places such as Hong Kong had almost 100% mass masking since the end of January and seen good outbreak control. These challenges might be amplified during difficult times such as the COVID-19 pandemic. For example, some Asian international students have reported experiencing isolation and discrimination because they were perceived as potential COVID-19 carriers in their institution country (6). Wearing masks could also be stigmatized.

The current study focused on international students, some of whom stayed in their institution country and some of whom returned to their home country or region (which had a less severe outbreak or with outbreak better controlled) during the COVID-19 pandemic during the COVID-19 outbreaks. During the survey period (from April 28, 2020 through May 12, 2020), the COVID-19 outbreaks were escalating, with average daily increases of 4,681 and 28,185 confirmed COVID-19 cases per day, and a total of 223,064 and 1,322,054 confirmed cases on May 12, 2020 in the UK and USA, respectively (10). In Hong Kong, to where most of the students returned, the situation was under control with zero to four local cases per day during the study period (11). Owing to the escalating outbreaks in their institution countries, many students had returned to their home country or region where the outbreaks were perceived to be under better control.

Since major university destinations for international students such as the UK and the USA had more serious pandemic outbreaks with strict lockdown measures that may have impeded normal access to social support from family, friends, and universities, we hypothesized that international university students who stayed in their institution country (stayers) would have higher stress from COVID-19-related stressors (including individual, interpersonal, and environmental factors), which were associated with higher negative mental health impacts (perceived stress, and symptoms of anxiety, depression, and insomnia), than those who returned to their home country or region (returnees). We also hypothesized that compared with males, females would have more adverse mental health impacts, since females might experience higher stress from COVID-19-related stressors.

The objectives of this study were to (i) investigate whether stayers face more or less stress from COVID-19-related stressors and mental health problems than returnees, (ii) examine the differences in COVID-19-related stressors and mental health impacts between males and females, (iii) explore the association between resilience and family functioning and the mental health impacts of COVID-19 on students, and (iii) identify the COVID-19-related stressors and coping strategies that predict students' perceived stress level [Perceived Stress Scale-10 (PSS-10)], severity of anxiety and depression symptoms [Patient Health Questionnaire-4 (PHQ-4)], and severity of insomnia symptoms [Insomnia Severity Index (ISI)].

## Methods

### Study Design and Participants

We conducted a cross-sectional online questionnaire survey to collect information on the mental health impacts of the COVID-19 outbreak, resilience, family functioning, and stress coping strategies in international students studying abroad. Written informed consent was obtained before answering the survey. Ethics approval was granted by the Institutional Review Board of The University of Hong Kong/Hospital Authority Hong Kong West Cluster (reference number: UW20-298). The study was registered with the National Institutes of Health (identifier number: NCT04365361).

The inclusion criteria targeted full-time international university student aged 18 years or older studying abroad in the UK or USA. Written informed consent was obtained from all respondents.

### Procedures

The online questionnaire was distributed through an anonymous link with an exponential non-discriminative snowball sampling strategy. Considering time sensitivity, snowball sampling was a cost-effective and efficient method to reach our study population, which may have been difficult to sample otherwise (12). The link was first disseminated through the WhatsApp messaging platform to university students studying in Hong Kong or overseas. These students were encouraged to forward the survey link to their friends. To protect against duplicate responses, the online questionnaire was set up such that browser cookies would prevent respondents from taking the survey a second time using the same browser. Upon completion of the questionnaire, respondents received automatically computed scores with brief interpretations and explanations for scales included in the questionnaire in order to promote mental health awareness. No incentives were given to respondents, but links for reliable information on COVID-19 (e.g., link to the World Health Organization website) and telephone numbers for seeking help, support, or further information were provided.

### Measurement Tools

A self-administered, anonymous questionnaire based on components of the transactional model of stress and adaptive coping was used to collect respondents' demographic characteristics, academic program, stress from COVID-19-related stressors, mental health impacts, resilience, family functioning, and stress coping strategies (13).

#### Academic Program Characteristics

Respondents were asked to indicate (i) their institution country, (ii) whether they were full-time or part-time students, (iii) whether they were final-year students, (iv) whether their academic program included a practicum component, and (v) whether the program was medical orZ health related.

#### Coronavirus Disease 2019-Related Stressors

Respondents were asked to indicate how stressful they found nine possible COVID-19-related stressors, under three groups: individual (academic attainment, personal health, and health of friends or family), interpersonal (lack of social support and prejudiced attitude or behavior of others), and environmental (uncertainties about the academic program, changes in teaching/learning format, the economic impact of COVID-19, and availability of reliable COVID-related information). Responses were made on a five-point Likert scale: “1 = not at all stressful,” “2 = mildly stressful,” “3 = moderately stressful,” “4 = very stressful,” and “5 = extremely stressful”.

#### Perceived Stress Scale -10

The ten-item Perceived Stress Scale -10 (PSS-10) was used to assess perceived stress by asking respondents how often they had certain thoughts and feelings during the past month. Scores ranged from 0 to 40, with cutoffs for low (0–13), moderate (14–26), and high (27–40) perceived stress. Cronbach's alpha of 0.83 was reported (14).

#### Patient Health Questionnaire-4

The four-item Patient Health Questionnaire-4 (PHQ-4) was used as an ultra-brief screening for symptoms of anxiety and depression. Scores ranged from 0 to 12, with cutoffs for normal (0–2), mild (3–5), moderate (6–8), and severe (9–12) anxiety and depression symptoms. Cronbach's alpha of 0.85 was reported (15).

#### Insomnia Severity Index

The seven-item Insomnia Severity Scale (ISI) was used to assess the severity of insomnia symptoms. Scores ranged from 0 to 28, with cutoffs for no clinically significant insomnia (0–7), subthreshold insomnia (8–14), moderate clinical insomnia (15–21), and severe clinical insomnia (22–28). Cronbach's alpha of 0.83 was reported (16).

#### Brief Assessment of Family Functioning Scale

The three-item Brief Assessment of Family Functioning Scale (BAFFS) was used to assess respondents' family functioning. Scores ranged from 4 to 12, with higher scores indicating greater family distress. Cronbach's alpha of 0.71 was reported (17, 18).

#### Connor–Davidson Resilience Scale-2

The two-item Connor–Davidson Resilience Scale-2 (CD-RISC-2) was used to assess adaptability and resilience. Scores ranged from 0 to 8, with higher scores indicating better adaptability and resilience. Cronbach's alpha of 0.79 was reported (19).

#### Coping Strategies

Respondents were asked to indicate, from a list, the coping strategies they had utilized within the past month to relieve COVID-19-related stress. The items included listening to music, eating or cooking, video or mobile gaming, seeking support from family and friends, browsing the web, positive thinking, exercise, religious support, and meditation.

### Statistical Analysis

All quantitative statistical analyses were performed with SPSS for Windows (version 23.0). Chi-square test was used to examine the differences in the demographic characteristics and academic programs of the stayers and the returnees. Respondents who did not complete the questionnaires were excluded.

To control for potential confounders, the analyses were adjusted for sex (male vs. female), age group (18 to 25 vs. 25 years or older), ethnicity (Asian vs. non-Asian), country or region of residence (Hong Kong vs. others), country of study (UK vs. USA), education program level (undergraduate vs. postgraduate), program year (final year vs. non-final year), and field of study (medical or health-related vs. others).

Linear regression was used to examine the differences in stress from COVID-19-related stressors, mental health impacts [perceived stress levels (PSS-10), severity of anxiety and depression symptoms (PHQ-4), and severity of insomnia symptoms (ISI)], resilience (CD-RISC-2), and family functioning (BAFFS) between the stayers and returnees and between males and females. Binary multivariable logistic regression was used to examine the differences in the severity of perceived stress (“low” vs. “moderate to high”), anxiety and depression symptoms (“normal to mild” vs. “moderate to severe”), and insomnia symptoms (“none to threshold” vs. “moderate to severe”), between the stayers and returnees and between males and females.

For the total sample, analyses included forced entry of the above potential confounders, and respondents' return status (returnees vs. stayers). The linear relationship of mental health impacts with resilience and family functioning was examined using partial correlation coefficients.

Forward stepwise multiple linear regression was used to identify predictors of students' mental health impacts. First, the interaction effect between students' return status and sex was examined by forcing the return status by sex interaction term into the models. The dependent variables included perceived stress level, severity of anxiety and depression symptoms, and severity of insomnia symptoms. Academic program characteristics, COVID-19-related stressors, resilience, family functioning, and coping strategies were considered as independent variables influencing mental health impacts. If the interaction term (return status by sex) was not statistically significant, forward stepwise regression analysis was performed without the interaction term. The change in adjusted *R*^2^ was calculated with the removal of each significant variable from the model. All tests were two-sided, with *P* < 0.05 indicating statistical significance and *P* < 0.1 to *P* ≥ 0.5 indicating marginal statistical significance.

## Results

### Recruitment

**Figure 1:**
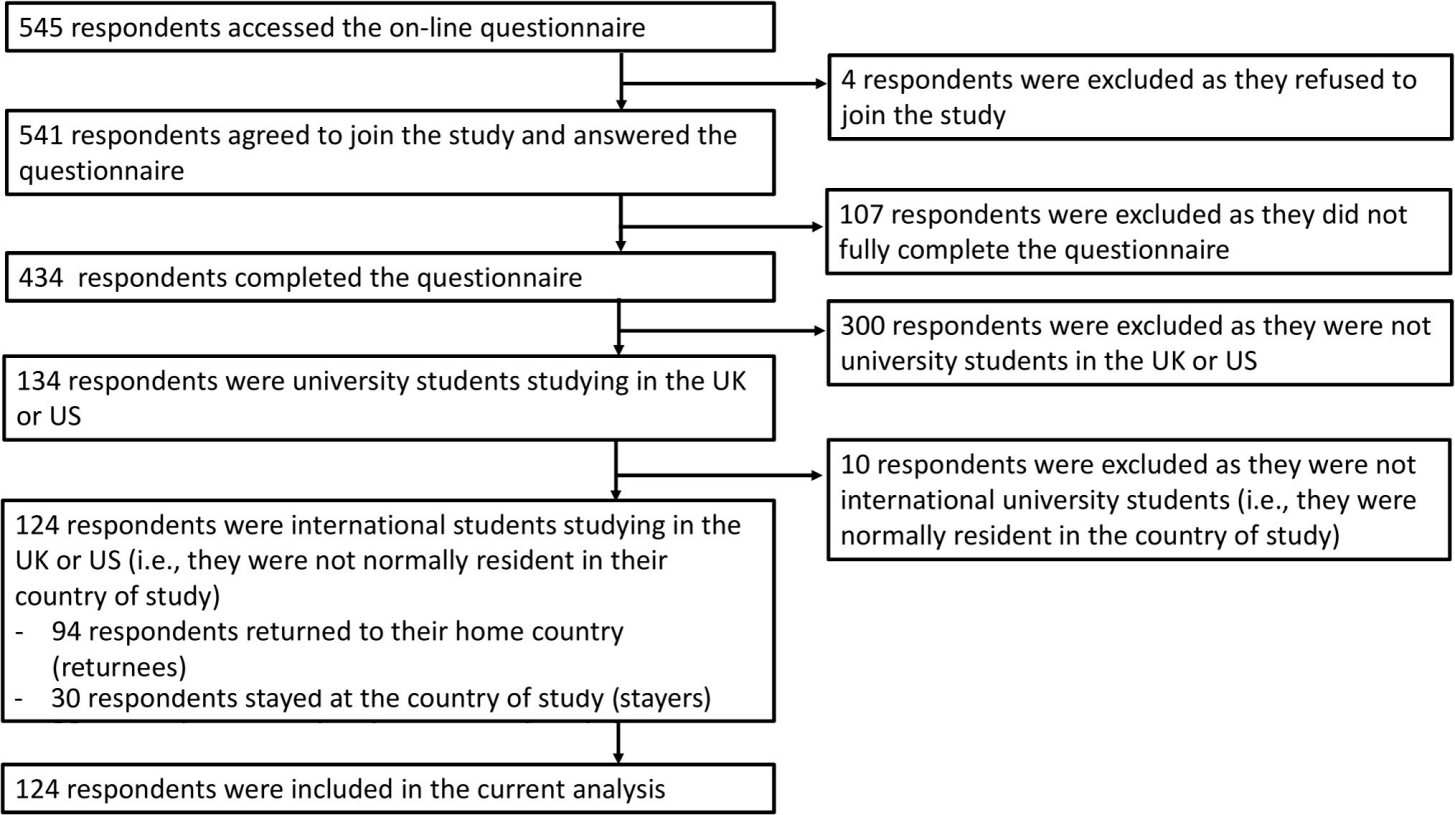
Recruitment flow chart.

A total of 545 students accessed the online survey during study period, and 541 agreed to join; 107 students who did not complete the questionnaire, 300 students not studying in the UK or USA, and 10 students who were not international students were excluded. Thus, the current analysis included 124 full-time international university students studying in the UK or USA who completed the questionnaire (Figure 1).

### Participants

Of the 124 students included, 36.3% were males, 86.3% were aged 18–25 years, and 41.9% were final-year students; 77.4% were pursuing a bachelor's program, 46.0% were pursuing medical or health-related programs, and 53.2% were in programs with practicum component; 75.8% had returned to their home country or region for reasons related to COVID-19. Among the returnees, 81% had returned to their home country or region on or before the end of March. Table 1 shows that compared with stayers, more returnees were younger, studying in the UK, undergraduates, from Hong Kong, in their non-final year, and in medical or health-related fields.

**Table 1:** Characteristics of international students in the UK and USA who returned to their home country or region (returnees) and those who stayed in their institution country (stayers).

	**All**	**Returnees**	**Stayers**	***P*-value**
	***n* = 124**	***n* = 94**	***n* = 30**	
	***n* (%)**	***n* (%)**	***n* (%)**	
**Sex**				
Males	45 (36.3)	33 (35.1)	12 (40.0)	0.63
Females	79 (63.7)	61 (64.9)	18 (60.0)	
**Age group**				
18–25 years	107 (86.3)	87 (92.6)	20 (66.7)	<0.001^***^
25 years or above	17 (13.7)	7 (7.4)	10 (33.7)	
**Ethnicity**				
Asian	116 (93.5)	89 (94.7)	27 (90.0)	0.36
Non-Asian	8 (6.3)	5 (5.3)	3 (10.0)	
**Country of study**				
UK	115 (92.7)	91 (96.8)	24 (80.0)	0.006^**^
USA	9 (7.3)	3 (3.2)	6 (20.0)	
**Country or region of residence**				
Hong Kong, China	100 (80.6)	84 (89.4)	16 (53.3)	<0.001^***^
Others	24 (19.4)	10 (10.6)	14 (46.7)	
**Education program level**				
Undergraduate	96 (77.4)	83 (88.3)	13 (43.3)	<0.001^**^
Postgraduate	28 (22.6)	11 (11.7)	17 (56.7)	
**Program year**				
Final year	52 (41.9)	32 (34.0)	20 (66.7)	0.002^**^
Non-final year	72 (58.1)	62 (66.0)	10 (33.3)	
**Program with practicum component**				
Yes	66 (53.2)	53 (56.4)	13 (43.3)	0.21
No	58 (46.8)	41 (43.6)	17 (56.7)	
**Field of study**				
Medical or health-related	57 (46.0)	50 (53.2)	7 (23.3)	0.004^**^
Other	67 (54.0)	44 (46.8)	23 (76.7)	

*From chi-square test or independent t-test*;

*** *P < 0.001*,

** *P < 0.01*.

### Coronavirus Disease 2019-Related Stressors

Table 2 shows that compared with returnees, stayers reported significantly higher levels of stress related to personal health {β [95% confidence interval (CI)]: 0.560 (0.146, 0.975), *P* = 0.01, Cohen's d: 0.57} and lack of social support [β (95% CI): 1.206 (0.752, 1.660), *P* < 0.001, Cohen's d: 1.11], with moderate-to-large effect sizes. Stayers also had marginally significantly higher stress related to the availability of reliable information on COVID-19 [β (95% CI): 0.426 (−0.034, 0.887), *P* = 0.07, Cohen's d: 0.39] and the prejudiced attitude or behavior of others [β (95% CI): 0.413 (−0.058, 0.844), *P* = 0.09, Cohen's d: 0.38] than returnees with small-to-moderate effect sizes.

**Table 2:** Stress levels from coronavirus disease 2019 (COVID-19)-related stressors in the total student sample and subgroups.

			**Return status**				**Sex**			
	**All *n* = 124**	**Students with high stress**^a^****	**Returnees *n* = 94**	**Stayers *n* = 30**	**Adjusted**^b^****		**Males *n* = 45**	**Females *n* = 79**	**Adjusted^c^**	
	**Mean ± SD**	***n* (%)**	**Mean ± SD**	**Mean ± SD**	**β (95% CI)**	**Effect size**^d^****	**Mean ± SD**	**Mean ± SD**	**β (95% CI)**	**Effect size**^d^****
**Individual factors**										
Academic attainment	3.19 ± 1.22	50 (40.3)	3.10 ± 1.26	3.47 ± 1.04	0.184 (−0.457, 0.825)	0.12	3.00 ± 1.28	3.29 ± 1.18	0.299 (−0.168, 0.767)	0.24
Personal health	1.85 ± 0.87	2 (1.6)	1.71 ± 0.77	2.27 ± 0.79	0.560 (0.146, 0.975)^**^	0.57	1.71 ± 0.79	1.92 ± 0.81	0.265 (−0.037, 0.568)^†^	0.28
Health of family or friends	2.04 ± 0.90	7 (5.2)	1.98 ± 0.92	2.23 ± 0.82	0.342 (−0.134, 0.818)	0.30	1.93 ± 0.86	2.10 ± 0.91	0.232 (−0.115, 0.578)	0.25
**Interpersonal factors**										
Lack of social support	1.81 ± 1.03	11 (8.9)	1.50 ± 0.65	2.80 ± 1.35	1.206 (0.752, 1.660)^***^	1.11	1.93 ± 1.1	1.75 ± 0.98	−0.179 (−0.510, 0.152)	0.35
Prejudiced attitude or behavior of others	1.77 ± 0.91	7 (5.6)	1.64 ± 0.80	2.17 ± 1.12	0.413 (−0.058, 0.844)^†^	0.38	1.82 ± 0.92	1.73 ± 0.92	−0.097 (−0.440, 0.247)	0.10
**Environmental factors**										
Uncertainties about academic program	2.85 ± 1.28	42 (34.0)	2.74 ± 1.24	3.17 ± 1.37	0.443 (−0.212, 1.099)	0.28	2.51 ± 1.31	3.04 ± 1.22	0.578 (0.099, 1.056)^*^	0.45
Changes in teaching/learning format	2.45 ± 1.24	28 (22.5)	2.28 ± 1.21	3.0 ± 1.17	0.418 (−0.210, 1.047)	0.28	2.29 ± 1.16	2.54 ± 1.28	0.316 (−0.143, 0.774)	0.26
Economic impact of COVID-19	2.29 ± 1.10	20 (16.1)	2.22 ± 1.09	2.50 ± 1.14	−0.005 (−0.577, 0.566)	0.01	2.16 ± 1.19	2.37 ± 1.05	0.182 (−0.235, 0.599)	0.16
Availability of reliable COVID-19 related information	1.85 ± 0.96	8 (6.5)	1.71 ± 0.81	2.30 ± 1.24	0.426 (−0.034, 0.887)^†^	0.39	1.96 ± 1.09	1.80 ± 0.88	−0.113 (−0.449, 0.223)	0.13

*Higher scores indicate higher stress levels; range: 1 = not at all stressful, 2 = mild stressful, 3 = moderately stressful, 4 = very stressful, and 5 = extremely stressful*.

a *Students with high stress refers those students rated the stress as either “4 = very stressful” or “5 = extremely stressful”*.

b *Between-group differences of variables adjusted for sex, age group, ethnicity, country or region of residence, country of study, education program level, program year, and field of study*.

c *Between-group differences of variables adjusted for return status, age group, ethnicity, country or region of residence, and country of study, education program level, program year, and field of study*.

d *Effect size (Cohen's d): small = 0.20, medium = 0.50, and large = 0.80*.

*** *P < 0.001*,

** *P < 0.01*,

* *P < 0.05*,

† *P < 0.1*.

Compared with males, females reported significantly higher stress related to uncertainties about academic program [β (95% CI): 0.578 (0.099, 1.056), *P* = 0.02, Cohen's d: 0.45] with small effect size and marginally significantly higher stress related to personal health [β (95% CI): 0.265 (−0.037, −0.568), *P* = 0.09 Cohen's d: 0.28].

### Mental Health Impacts

Of all students, 84.7% had moderate-to-high perceived stress, 12.1% had moderate-to-severe symptoms of anxiety and depression, and 17.7% had moderate-to-severe symptoms of insomnia (Table 3). Perceived stress level, severity of symptoms of anxiety and depression, and severity of symptoms of insomnia were significantly associated with each other (all *P* < 0.001) and stress from COVID-19-related stressors (Table 4).

**Table 3:** Levels and severity of mental health impacts, resilience, and family functioning in the total student sample and subgroups.

		**Return status**				**Sex**			
	**All *n* = 124**	**Returnees *n* = 94**	**Stayers *n* = 30**	**Adjusted**^a^****		**Males *n* = 45**	**Females *n* = 79**	**Adjusted**^b^****	
**Levels of mental health impacts, resilience, and family functioning**	**Mean ± SD**	**Mean ± SD**	**Mean ± SD**	**β (95% CI)**	**Effect size**^c^****	**Mean ± SD**	**Mean ± SD**	**β (95% CI)**	**Effect size**^c^****
Perceived stress level (PSS-10)^1^	19.9 ± 6.3	19.1 ± 6.1	22.6 ± 6.2	4.039 (0.816, 7.261)^*^	0.52	18.8 ± 6.9	20.6 ± 5.8	2.212 (−0.140, 4.564)^†^	0.35
Anxiety and depression symptoms (PHQ-4)^2^	3.2 ± 1.9	3.1 ± 1.9	3.6 ± 2.0	0.275 (−0.721, 1.272)	0.12	3.0 ± 2.1	3.4 ± 1.8	0.288 (−0.439, 1.016)	0.15
Insomnia symptoms (ISI)^3^	8.6 ± 5.7	7.6 ± 5.2	11.8 ± 6.1	3.087 (0.262, 5.912)^*^	0.45	7.4 ± 5.8	9.3 ± 5.6	1.223 (−0.838, 3.285)	0.22
Resilience (CD-RISC-2)^4^	5.1 ± 1.6	5.1 ± 1.6	5.0 ± 1.7	0.149 (−0.696, 0.995)	0.07	5.6 ± 1.5	4.8 ± 1.6	−0.717 (−1.334, −0.100)^*^	0.43
Family functioning (BAFFS)^5^	5.8 ± 1.7	5.9 ± 1.7	5.7 ± 1.7	0.313 (−0.607, 1.233)	0.12	6.1 ± 1.8	5.6 ± 1.6	−0.427 (−1.099, 0.244)	0.23
**Severity of mental health impacts**	***n*** **(%)**	***n*** **(%)**	***n*** **(%)**	**OR (95% CI)^d^**		***n*** **=** **45**	***n*** **=** **79**	**OR (95% CI)^d^**	
Perceived stress level (PSS-10)^1^									
Low (reference)	19 (15.3)	16 (17.0)	3 (10.0)			10 (22.2)	9 (11.4)		
Moderate to high	105 (84.7)	78 (83.0)	27 (90.0)	2.12 (0.39, 11.60)		35 (77.8)	70 (88.6)	2.08 (0.72, 5.60)	
Anxiety and depression symptoms (PHQ-4)^2^									
Normal to mild (reference)	109 (87.9)	84 (89.4)	25 (83.3)			39 (86.7)	70 (88.6)		
Moderate to severe	15 (12.1)	10 (10.6)	5 (16.7)	1.41 (0.29, 6.93)		6 (13.3)	9 (11.4)	0.82 (0.25, 2.72)	
Severity of insomnia symptoms (ISI)^3^									
None to threshold (reference)	102 (82.3)	83 (88.3)	19 (63.3)			38 (84.4)	64 (81.0)		
Moderate to severe	22 (17.7)	11 (11.7)	11 (36.7)	2.91 (0.76, 11.10)		7 (15.6)	15 (19.0)	1.03 (0.322, 3.30)	

1 *PSS-10: 10-item Perceived Stress Scale to measure perceived stress level; higher scores indicate higher stress level; range, 0–40; low, 0–13; moderate to high, 14–40*.

2 *PHQ-4: 4-item Patient Health Questionnaire to screen for anxiety and depression symptoms; higher scores indicate more severe symptoms; range, 0–12; normal to mild, 0–5; moderate to severe, 6–12*.

3 *ISI: 7-item Insomnia Severity Index to assess the severity of insomnia symptoms; higher scores indicate more severe symptoms; range, 0–28; none to threshold, 0–14; moderate to severe, 15–28*.

4 *CD-RISC-2: 2-item version of the Connor–Davidson Resilience Scale to assess resilience; higher scores indicate better adaptability; range, 0–8*.

5 *BAFFS: 3-item Brief Assessment of Family Functioning Scale to evaluate family functioning; higher scores indicate greater distress; range, 4–12*.

a *Between-group differences of variables adjusted for sex, age group ethnicity, country or region of residence, country of study, education program level, program year, and field of study*.

b *Between-group differences of variables adjusted for return status, age group, ethnicity, country or region of residence, country of study, education program level, program year, and field of study*.

c *Effect size (Cohen's d): small = 0.20, medium = 0.50, and large = 0.80*.

d *OR (95% CI) = odds ratio (95% confidence interval)*.

* *P < 0.05*,

† *P < 0.1*.

**Table 4:** Association between mental health impacts and coronavirus disease 2019 (COVID-19)-related stressors, coping factors, and strategies.

	**Perceived stress level (PSS-10)**		**Severity of anxiety and depression symptoms (PHQ-4)**		**Severity of insomnia symptoms (ISI)**	
	***r***	***P*-value**	***r***	***P*-value**	***r***	***P*-value**
**MENTAL HEALTH**						
Perceived stress level (PSS-10)	–	–	0.477	<0.001^***^	0.489	<0.001^***^
Severity of anxiety and depression symptoms (PHQ-4)	0.477	<0.001^***^	–	–	0.444	<0.001^***^
Severity of insomnia symptoms (ISI)	0.489	<0.001^***^	0.444	<0.001^***^	–	–
**COVID-19 RELATED STRESSORS**						
**Individual factors**						
Academic attainment	0.532	<0.001^***^	0.344	<0.001^***^	0.245	<0.001^***^
Personal health	0.268	<0.001^***^	0.356	<0.001^***^	0.364	<0.001^***^
Health of family or friends	0.317	<0.001^***^	0.319	<0.001^***^	0.277	0.011^**^
**Interpersonal factors**						
Lack of social support	0.404	<0.001^***^	0.332	<0.001^***^	0.370	<0.001^***^
Prejudiced attitude or behavior of others	0.276	0.002^**^	0.297	0.002^**^	0.200	0.026^*^
**Environmental factors**						
Uncertainties about academic program	0.438	<0.001^***^	0.326	<0.001^***^	0.278	0.002^**^
Changes in teaching/learning format	0.477	<0.001^***^	0.369	<0.001^***^	0.258	0.004^**^
Economic impact of COVID-19	0.195	0.03^*^	0.296	0.001^**^	0.122	0.18
Availability of reliable COVID-19 related information	0.344	<0.001^***^	0.379	<0.001^***^	0.241	0.007^**^
**Coping factors**						
Resilience	−0.495	<0.001^***^	−0.453	<0.001^***^	−0.297	<0.001^***^
Family functioning	0.238	0.008^**^	0.216	0.016^*^	0.211	0.019^*^
**Coping strategies**						
Listening to music	−0.009	0.92	0.061	0.50	−0.093	0.30
Eating or cooking	0.147	0.10	0.218	0.015^*^	0.215	0.017^*^
Video/mobile gaming	0.020	0.83	−0.022	0.81	0.062	0.50
Seeking support from family/friends	−0.041	0.65	−0.018	0.84	−0.213	0.018^*^
Browsing the web	0.017	0.85	0.043	0.639	−0.010	0.910
Positive thinking	−0.176	0.049^*^	−0.142	0.116	−0.209	0.020^*^
Exercise	−0.146	0.11	−0.194	0.031^*^	−0.031	0.73
Religious support	−0.076	0.40	−0.037	0.680	−0.050	0.58
Meditation	0.008	0.93	−0.066	0.47	−0.067	0.46

*** *P < 0.001*,

** *P < 0.01*,

* *P < 0.05*.

Compared with returnees, stayers had significantly higher perceived stress [PSS-10: 22.6 ± 6.2 vs. 19.1 ± 6.1, β (95% CI): 4.039 (0.816, 7.261), *P* = 0.02, Cohen's d: 0.52] and more severe insomnia symptoms [ISIs: 11.8 ± 6.1 vs. 7.6 ± 5.2, β (95% CI): 3.087 (0.262, 5.912), *P* = 0.03, Cohen's d: 0.46], with moderate effect sizes (Table 3). No significant difference in severity of anxiety and depression symptoms (PHQ-4) between returnees and stayers was found.

Compared with males, females reported marginally significantly higher perceived stress [PSS-10: 20.6 ± 5.8 vs. 18.8 ± 6.9, β (95% CI): 2.212 (−0.140, 4.564), *P* = 0.07, Cohen's d: 0.35] with small effect size. However, no significant difference in severity of anxiety and depression symptoms and insomnia symptoms between males and females was found.

### Coronavirus Disease 2019-Related Stressors Predicting Mental Health Impacts

Table 4 shows that stress from all COVID-19-related stressors was significantly associated with perceived stress level, severity of anxiety and depression symptoms, and severity of insomnia symptoms (all *P* < 0.05), with the exception of stress from the economic impact of COVID-19, which was not significantly associated with the severity of insomnia symptoms (*r* = 0.122, *P* = 0.18).

For COVID-19-related stressors predicting mental health impacts, no statistically significant interaction effects of return status by sex were found (return status by sex interaction term: PSS-10, *P* = 0.18; PHQ-4, *P* = 0.07; ISI, *P* = 0.22). Table 5 shows that stress related to academic attainment (adjusted *R*^2^ = 23.4%) was the most important predictor of perceived stress level (PSS-10), followed by lack of social support and uncertainties about academic program. Stress related to the changes in teaching/learning format (adjusted *R*^2^ = 9.9%) was the most important predictor of the severity of anxiety and depression symptoms (PHQ-4), followed by health of family and friends and availability of reliable information on COVID-19. The most important predictor of the severity of insomnia symptoms (ISI) was stress related to personal health (adjusted *R*^2^ = 5.7%), followed by uncertainties about the academic program.

**Table 5:** Coronavirus disease 2019 (COVID-19)-related stressors as predictors of mental health impacts identified by forward stepwise multiple regression analysis (*n* = 124).

	**Change in adjusted *R*^2^**	**Estimate (SE)**	***P*-value**
**Dependent variable 1: perceived stress level (PSS-10)^1^Adjusted** ***R*****^2^** **=** **38.0%**			
Sex, age group, ethnicity, country of study, country or region of residence, return status, education program level, program year, and field of study	8.0%	–	–
Academic attainment	23.4%	1.938 (0.452)	<0.001^***^
Lack of social support	5.0%	1.781 (0.552)	0.002^**^
Uncertainties about academic program	1.6%	0.871 (0.437)	0.049^*^
**Dependent variable 2: severity of anxiety and depression symptoms (PHQ-4)^2^Adjusted** ***R*****^2^** **=** **23.2%**			
Sex, age group, ethnicity, country of study, country or region of residence, return status, education program level, program year, and field of study	3.9%	–	–
Changes in teaching/learning format	9.9%	0.374 (0.141)	0.009^**^
Health of family/friends	7.1%	0.529 (0.180)	0.004^**^
Availability of reliable information related to COVID-19	2.3%	0.404 (0.196)	0.041^**^
**Dependent variable 3: severity of insomnia symptoms (ISI)**^3^**Adjusted** ***R*****^2^** **=** **22.9%**			
Sex, age group, ethnicity, country of study, country or region of residence, return status, education program level, program year, and field of study	14.6%	–	–
Personal health	5.7%	1.738 (0.610)	0.005^**^
Uncertainties about academic program	2.6%	0.846 (0.385)	0.030^*^

1 *PSS-10: 10-item Perceived Stress Scale to measure perceived stress level; higher scores indicate higher stress level; range, 0–40*.

2 *PHQ-4: 4-item Patient Health Questionnaire to screen for anxiety and depression symptoms; higher scores indicate more symptoms; range, 0–12*.

3 *ISI: 7-item Insomnia Severity Index to assess the severity of insomnia symptoms; higher scores indicate more symptoms; range, 0–28. Forward stepwise multiple linear regression was used. The interaction effect between students' return status and sex was examined by forcing the interaction term of return status by sex, return status, sex, age group, ethnicity, country or region of residence, country of study, education program level, program year, and field of study into the regression models for adjustment of confounders. If the interaction term (return status by sex) was not statistically significant, the forward stepwise regression analysis was performed without the interaction term*.

*Considered independent variables included COVID-19-related stressors, including personal health, health of friends or family, academic attainment, prejudiced attitude or behavior of others, lack of social support, changes in teaching/learning format, uncertainties about academic program, availability of reliable information related to COVID-19, and economic impact of COVID-19*.

*Since the interaction term in the above analyses was not statistically significant, the above-presented models did not include the interaction term, and the change in adjusted R^2^ was calculated from removal of each significant variable from the model*.

*** *P < 0.001*,

** *P < 0.01*,

* *P < 0.05*.

### Resilience, Family Functioning, and Mental Health Impacts

Resilience was significantly negatively correlated with lower perceived stress level (PSS-10: *r* = −0.526, *P* < 0.001), severity of anxiety and depression symptoms (PHQ-4: *r* = −0.467, *P* < 0.001), and severity of insomnia symptoms (ISI: *r* = −0.328, *P* = 0.001) (Table 4). Compared with males, females reported significantly lower resilience [CD-RISC-2: 5.6 ± 1.5 vs. 4.8 ± 1.6, β (95% CI): −0.717 (−1.334, −0.100), *P* = 0.02, Cohen's d: 0.43] with small effect size. However, there was no significant difference in resilience between stayers and returnees (Table 3).

Family functioning (BAFFS; higher scores indicate greater distress) was significantly correlated with higher perceived stress level (PSS-10: *r* = 0.258, *P* = 0.008), severity of anxiety and depression symptoms (PHQ-4: *r* = 0.234, *P* = 0.0161), and severity of insomnia symptoms (ISI: *r* = 0.251, *P* = 0.02) (Table 4). No significant difference in resilience between stayers and returnees, as well as between males and females, was found (Table 3).

### Resilience and Coping Strategies Predicting Mental Health Impacts

**Figure 2:**
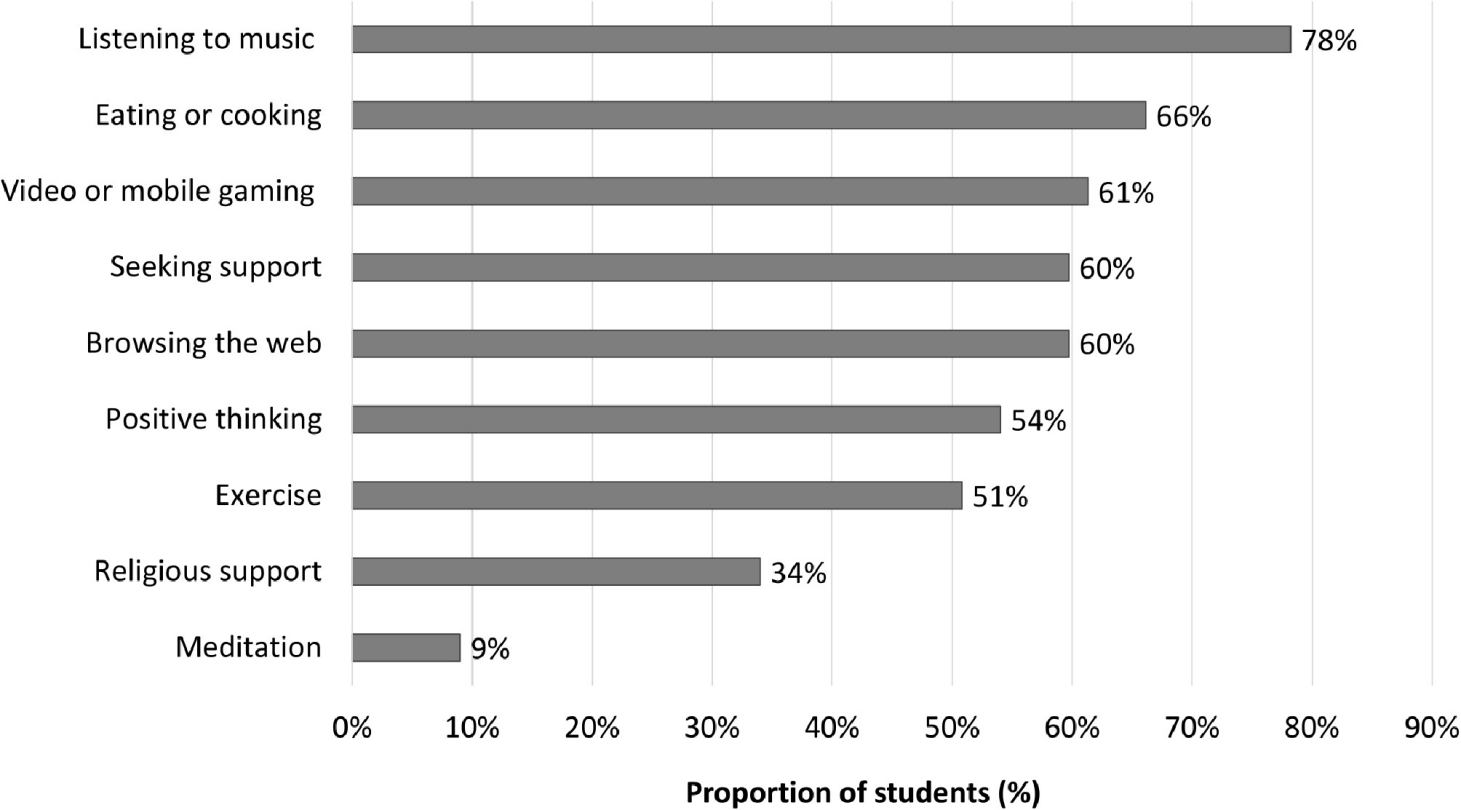
Coping strategies in response to coronavirus disease 2019 (COVID-19) for the total student sample.

The top three most commonly used coping strategies among students during the COVID-19 pandemic were listening to music (78%), eating or cooking (66%), and video or mobile gaming (61%) (Figure 2).

Table 4 shows that eating or cooking was significantly positively associated with severity of anxiety and depression symptoms and insomnia symptoms. Positive thinking was significantly negatively associated with perceived stress and severity of insomnia symptoms. Exercise was significantly positively associated with severity of anxiety and depression symptoms (all *P* < 0.05).

The return status by sex interaction term was not significant (interaction term: PSS-10, *P* = 0.52; PHQ-4, *P* = 0.39; ISI, *P* = 0.52) and was not included in the forward stepwise multiple regression analysis.

Table 6 shows that resilience was the most important predictor of mental health impacts [perceived stress (PSS-10), adjusted *R*^2^ = 27.0%; severity of anxiety and depression symptoms (PHQ-4), adjusted *R*^2^ = 20.6%; severity of insomnia symptoms (ISI), adjusted *R*^2^ = 9.5%]. Positive thinking, exercise, and seeking support from family and friends were coping strategies that were predictors of less severe mental health impacts.

**Table 6:** Resilience and coping strategies as predictors of mental health impacts identified by forward stepwise multiple regression analysis (*n* = 124).

	**Change in adjusted *R***^2^****	**Estimate (SE)**	***P*-value**
**Dependent variable 1: perceived stress level (PSS-10)^1^Adjusted** ***R*****^2^** **=** **37.8%**			
Sex, age group, ethnicity, country of study, country or region of residence, return status, education program level, program year, and field of study	8.0%	–	–
Resilience (CD-RISC-2)	27.0%	−2.058 (0.294)	<0.001^***^
Positive thinking	2.8%	−2.251 (0.908)	0.015^*^
**Dependent variable 2: severity of anxiety and depression symptoms (PHQ-4)^2^Adjusted** ***R*****^2^** **=** **33.2%**			
Sex, age group, ethnicity, country of study country or region of residence, return status, education program level, program year, and field of study	3.9%	–	–
Resilience (CD-RISC-2)	20.6%	−0.538 (0.094)	<0.001^***^
Eating or cooking	4.1%	0.977 (0.327)	0.003^**^
Exercise	2.5%	−0.643 (0.293)	0.030^*^
Positive thinking	2.1%	−0.605 (0.285)	0.036^*^
**Dependent variable 3: severity of insomnia symptoms (ISI)^3^Adjusted** ***R*****^2^** **=** **31.5%**			
Sex, age group, ethnicity, country of study country or region of residence, return status, education program level, program year, and field of study	14.6%	–	–
Resilience (CD-RISC-2)	9.5%	−1.097 (0.281)	<0.001^***^
Seeking support from family/friends	5.3%	−2.218 (0.966)	0.024^*^
Positive thinking	2.1%	−1.938 (0.912)	0.036^*^

1 *PSS-10: 10-item Perceived Stress Scale to measure perceived stress level; higher scores indicate higher stress level; range, 0–40*.

2 *PHQ-4: 4-item Patient Health Questionnaire to screen for anxiety and depression symptoms; higher scores indicate more symptoms; range, 0–12*.

3 *ISI: 7-item Insomnia Severity Index to assess the severity of insomnia symptoms; higher scores indicate more symptoms; range, 0–28. Forward stepwise multiple linear regression was used. The interaction effect between students' return status and sex was examined by forcing the interaction term of return status by sex, return status, sex, age group, ethnicity, country or region of residence, country of study, education program level, program year, and field of study into the regression models for adjustment of confounders. If the interaction term (return status by sex) was not statistically significant, the forward stepwise regression analysis was performed without the interaction term*.

*Considered independent variables included resilience (CD-RISC-2), family functioning (BAFFS), and coping strategies (listening to music, eating or cooking, video or mobile gaming, seeking support from family and friends, browsing the web, positive thinking, exercise, religious support, and meditation). Since the interaction term in the above analyses was not statistically significant, the above-presented models did not include the interaction term, and the change in adjusted R^2^ was calculated from removal of each significant variable from the model*.

*** *P < 0.001*,

** *P < 0.01*,

* *P < 0.05*.

## Discussion

Our study is the first study on stressors, coping strategies, and mental health impacts of COVID-19 in international students studying abroad. The findings showed that more than 80% of the students had moderate-to-high perceived stress. Stayers had higher stress related to personal health and lack of social support, perceived stress (PSS-10), and more ISIs than returnees; and females had higher stress related to uncertainties about the academic program and lower resilience than males.

In the sample, stress related to academics (e.g., personal academic attainment, uncertainties about the academic program, and changes in teaching/learning format), health (personal health and health of family and friends), availability of reliable COVID-19-related information, and lack of social support were predictive of higher perceived stress level and more severe anxiety and depression symptoms. Resilience and positive thinking were important coping strategies against negative mental health impacts.

A high proportion of students in our sample had moderate-to-severe perceived stress, which is consistent with the fact that university students often fall within the age range when common mental health problems are at their developmental peak (20). Students' stress may be exacerbated by experiences during the COVID-19 pandemic. In particular, Sahu noted that the closure of universities during the pandemic may pose monetary and mental health challenges to international students, among other challenges (21). We also found that females had higher stress related to uncertainties about academic program during the COVID-19 pandemic. This is consistent with other findings in the literature: Liu et al. found significantly greater increases in the prevalence and severity of posttraumatic symptoms in females, compared with males, during the initial phase of COVID-19 (22). Besides, significant bivariate associations were found between female and fear, as well as with mental health consequences (anxiety and depressive symptoms) (23).

In mass media, some international students have reported high stress related to difficulties obtaining air tickets at high prices, travel risks and restrictions, the quarantine process (for those planning to return home), and employment to cope with basic living expenses (for those planning to stay in their institution country) during the pandemic (24). We found that lack of social support was an important predictor of students' mental health. This is consistent with others' findings that social support is negatively correlated with adverse mental health impacts (25). Stayers reported higher stress than returnees. This difference could be explained by differences in the stayers and returnees' experiences: while stayers resided in their institution countries where the pandemic situation was not yet under control, information appeared unreliable, masking was stigmatized, and COVID-19-related policies were criticized as suboptimal, returnees could join their families in their home country or region. Returnees would have felt safer as COVID-19 was perceived to be under better control in their home country or region, while stayers would have experienced greater stress related to social isolation under mandatory lockdown in their institution countries amid unreliable information and controversial policies.

### Implications

Our work has important implications for academic institutions, clinical work, and public health. First, academic institutions, particularly those in the UK and USA, should increase their awareness of additional needs and potential mental health problems experienced by their students. International students already face stress related to the acculturation demands of studying abroad (26), and students' stress may be amplified during a public health crisis. Academic institutions should show more understanding and empathy toward these students, especially stayers. Course management needs to consider how best to relieve students' academic-related stress. Education and training for educators and mental health professionals on identifying risk factors and symptoms of mental distress from COVID-19 for better identification and management of students' mental health are advised.

Stayers may hesitate to seek support for emotional problems, fear stigma, and prefer to handle problems alone (27). Even if they are motivated to seek support, the lockdown regulations may have made the usual face-to-face student assistance and counseling services inaccessible. Educators, institutions, and mental health professionals need to proactively reach out to their students to understand their needs and provide assistance. Student support groups or counseling via e-platforms are urgently needed to help students alleviate mental health problems and provide social, psychological, and academic support.

Family functioning and resilience were reported to have a strong association with negative mental health impacts. Family functioning is one of the important aspects of the family environment, which affects the physical, social, and emotional health of individuals (28). Resilience is a protective factor that buffers from the effects of traumatic experience, which enhances individual adaptation and positively influences successful adaptation and coping (29). Besides, resilience, positive thinking, and exercise were identified as important coping strategies that predicted less severe mental health impacts in our study. Online mental health education and mindfulness-based interventions can help students enhance their resilience (30). Academic institutions should enact effective action plans to promote students' resilience through the official academic curriculum or unofficial student extracurricular activities that can be run under a lockdown or social distancing regulations.

In public health, frequent misinformation and rumors about viruses are common causes of distress (31). We have found that the availability of reliable information about COVID-19 was an important stressor for international students during the pandemic. Stronger collaboration between different parties, such as universities and health departments, could help with the timely delivery of precise and easy-to-understand information to the public, helping in turn with disease prevention and the implementation of precautionary measures.

### Limitations

Our study had several limitations. First, while snowball sampling was an effective strategy to recruit suitable respondents efficiently and allowed the study to capture valuable data at the height of the pandemic, sampling bias could have arisen from respondents forwarding the survey to peers with similar traits and characteristics (12) and the small sample size. The fact that no incentives were offered to respondents for their participation might explain the limited number of respondents recruited. We also wished to stop recruiting earlier so that our results could raise the alarm and call for remedial actions as soon as possible. Second and relatedly, the majority of the respondents (95%) were Asian, and our findings may not be applicable to other international students. Specifically, most of our respondents were students from Hong Kong studying in the UK. As the control measures for and the extent of the outbreaks of COVID-19 were different across countries, future studies should include international students across more countries and ethnicities. Finally, although the coping strategies included in our survey were strategies that may be popular among students, the list was not exhaustive, and popular strategies may not necessarily be the most effective strategies to protect against adverse mental health impacts. Further studies should investigate the efficacy of a more expansive series of coping strategies.

To conclude, the mental health impacts of COVID-19 on international students have been overlooked. We call on educators, academic institutions, and mental health professionals to provide appropriate support for their international students, particularly the stayers, during the pandemic.

## Data Availability Statement

The raw data supporting the conclusions of this article will be made available by the authors, without undue reservation.

## Ethics Statement

The studies involving human participants were reviewed and approved by the Institutional Review Board of The University of Hong Kong/Hospital Authority Hong Kong West Cluster (reference number: UW20-298). The patients/participants provided their written informed consent to participate in this study.

## Author Contributions

AL and LL led the conception and design of the survey, carried out the survey, and were responsible for interpreting the data and drafting the manuscript. AL and L-mH were involved in the statistical analysis of the data. AL, LL, T-hL, M-pW, MI, YF, TT-kL, and VL were closely involved in data interpretation and manuscript revision. All authors read and approved the final manuscript.

## Conflict of Interest

The authors declare that the research was conducted in the absence of any commercial or financial relationships that could be construed as a potential conflict of interest.
